# Large deletion at the *CDC73* gene locus and search for predictive markers of the presence of a *CDC73* genetic lesion

**DOI:** 10.18632/oncotarget.25067

**Published:** 2018-04-17

**Authors:** Lucia Anna Muscarella, Daniela Turchetti, Andrea Fontana, Filomena Baorda, Orazio Palumbo, Annamaria la Torre, Danilo de Martino, Renato Franco, Nunzia Simona Losito, Andrea Repaci, Uberto Pagotto, Luigia Cinque, Massimiliano Copetti, Maria Grazia Chiofalo, Luciano Pezzullo, Paolo Graziano, Alfredo Scillitani, Vito Guarnieri

**Affiliations:** ^1^ Laboratory of Oncology, IRCCS Casa Sollievo della Sofferenza Hospital, San Giovanni Rotondo 71013, Italy; ^2^ Medical Genetics, Sant’Orsola Malpighi Hospital, University of Bologna, Bologna 40138, Italy; ^3^ Unit of Biostatistics, IRCCS Casa Sollievo della Sofferenza Hospital, San Giovanni Rotondo 71013, Italy; ^4^ Medical Genetics, IRCCS Casa Sollievo della Sofferenza Hospital, San Giovanni Rotondo 71013, Italy; ^5^ ISBReMIT, Institute for Stem-cell Biology, Regenerative Medicine and Innovative Therapies, IRCCS Casa Sollievo della Sofferenza Hospital, San Giovanni Rotondo 71013, Italy; ^6^ Thoracic Surgery, IRCCS Casa Sollievo della Sofferenza Hospital, San Giovanni Rotondo 71013, Italy; ^7^ Pathology , Istituto Nazionale Tumori, Fondazione “G. Pascale”, Napoli 80131, Italy; ^8^ Endocrinology, Sant’Orsola Malpighi Hospital, University of Bologna, Bologna 40138, Italy; ^9^ Thyroid and Parathyroid Surgery Unit, Istituto Nazionale Tumori, Fondazione “G. Pascale”, Napoli 80131, Italy; ^10^ Pathology, IRCCS Casa Sollievo della Sofferenza Hospital, San Giovanni Rotondo 71013, Italy; ^11^ Endocrinology, IRCCS Casa Sollievo della Sofferenza Hospital, San Giovanni Rotondo 71013, Italy

**Keywords:** CDC73, large genomic deletion, HPT-JT, early onset

## Abstract

The Hyperparathyroidism with Jaw-Tumours syndrome is caused by mutations of the *CDC73* gene: it has been suggested that early onset of the disease and high Ca^2+^ levels may predict the presence of a *CDC73* mutation. We searched for large deletions at the *CDC73* locus in patients with: HPT-JT (nr 2), atypical adenoma (nr 7) or sporadic parathyroid carcinoma (nr 11) with a specific MLPA and qRT-PCR assays applied on DNA extracted from whole blood. A Medline search in database for all the papers reporting a *CDC73* gene mutation, clinical/histological diagnosis, age at onset, Ca^2+^, PTH levels for familial/sporadic cases was conducted with the aim to possibly identify biochemical/clinical markers predictive, in first diagnosis, of the presence of a *CDC73* gene mutation. A novel genomic deletion of the first 10 exons of the *CDC73* gene was found in a 3-generation HPT-JT family, confirmed by SNP array analysis. A classification tree built on the published data, showed the highest probability of having a *CDC73* mutation in subjects with age at the onset < 41.5 years (44/47 subjects, 93.6%, had the mutation). Whereas the lowest probability was found in subjects with age at the onset ≥ 41.5 years and Ca^2+^ levels <13.96 mg/dL (7/20 subjects, 35.0%, had the mutation, odds ratio = 27.1, *p* < 0.001). We report a novel large genomic CDC73 gene deletion identified in an Italian HPT-JT family. Age at onset < 41.5 ys and Ca^2+^ > 13.96 mg/dL are predictive for the presence of a CDC73 genetic lesion.

## INTRODUCTION

Parathyroid carcinoma (PC) is a rare, aggressive tumour for which, at present, neither effective nor surgical cure is available. It can be sporadic or part of the Hyperparathyroidism with Jaw Tumours (HPT-JT, MIM 145001).

HPT-JT is characterized by hyperparathyroidism, ossifying fibroma of the maxilla/jaw, parathyroid carcinoma, renal lesions (Wilms tumours or papillary renal cell carcinoma), uterine leiomiomata (BIBLIO). In HPT-JT, the hypercalcemia is due to a parathyroid lesion (a malignant PC in up to the 15% of cases) and it can range from mildly elevated levels, just above the upper limit to high severe life-threatening amounts demanding prompt intervention. The only curative approach for the PC is the surgery that, however, is not always supported by a robust first diagnosis.

Instead, in absence of pathognomic signs such as metastasis or local recurrences, the diagnosis at first observation of PC from hyperplasia or adenoma remains a challenge [1]. This is due to the extreme rarity of the tumour (0.005% of all cancers, less than 1% of primary hyperparathyroidism (PHPT) cases [1]), but also to the different clinical presentation of the PC: indeed it often presents with symptoms overlapping the ones induced by a classic benign parathyroid adenoma [2–4]; moreover rare cases of non-functioning PCs [2] or even in the setting of a normocalcemic PHPT [5], or of PCs masked by thyroid disorders [6] have been reported. Great effort has been made in search of possible immunohistochemistry (IHC) biomarkers for an ultimate post-surgical diagnosis [7]. However, this diagnostic default reflects on the surgery management, since an accurate first diagnosis would address towards a demolitive (in case of malignancy) or conservative (in case of a benign lesion) approach with a dramatic impact on the patient’s quality of life and long overall survival [4].

The identification of the main genetic driver of the hyperparathyroidism with Jaw Tumours (HPT-JT, MIM #145001) syndrome first [8] and then of familial and sporadic PC later [9], the CDC73 gene, helped in the molecular diagnosis and also in the follow up of subjects at risk to develop an aggressive parathyroid lesion, in case of familial forms. However, other molecular and/or clinical markers that could help the first diagnosis would be desirable.

The gene encodes for an ubiquitously expressed co-transcription factor, namely parafibromin, involved in RNA polymerase II transcription [10], chromatin remodelling [11] and p53 stabilization [12]. The vast majority of the CDC73 gene mutations are frameshift deletions/insertions or nonsense [13]. Recently, large genomic deletions at the CDC73 locus (1q32.1) have been also identified [14–19].

Interestingly, it was reported that CDC73 gene mutations (of which up to 1/3 could be large genomic deletions) [16] may affect subjects with hypercalcemic disorder, regardless of the diagnosis (PHPT, HPT-JT, Familial Isolated Hyperparathyrodism, FIHP, or sporadic PC) [16]. The same study suggested that early age at onset (AaO) of the disease and Ca^2+^ > 3 mM may indicate the presence of a CDC73 gene lesion (germline mutation/large deletion) [11]. However, a systematic search for large CDC73 gene deletions on selected patients negative for coding mutations was not performed, nor a better definition of the range of the AaO and Ca^2+^ levels, in which CDC73 mutation should be suspected, was attempted.

Here we report the search for large deletions at the CDC73 genetic locus on a selected survey of subjects affected by HPT-JT, sporadic or familial PC, atypical adenoma and previously resulted negative for coding sequence mutations. We applied two different methods: a novel ad hoc protocol of RT-qPCR for all the exons of the CDC73 gene and the commercial MLPA kit (MRC Holland). Positive subjects were also confirmed by SNP-array platform.

Moreover, we retrieved published data so far reported on subjects with a CDC73 gene mutations either as familial or sporadic cases. We collected all the data about gender, diagnosis, histology, age at onset, Ca^2+^ and PTH levels in order to determine whether some of these clinical/biochemical parameters could be considered as suggestive of a CDC73 genetic lesion and to define their cut-offs.

## RESULTS

### *CDC73* screening

PC4-5-11, JT1 and AA1-2-3-4 were previously screened for *CDC73* coding mutations and resulted negative [20]. Here we reported the screening on other patients that resulted negative as well.

### RTqPCR, MLPA and array CGH

**Figure 1:**
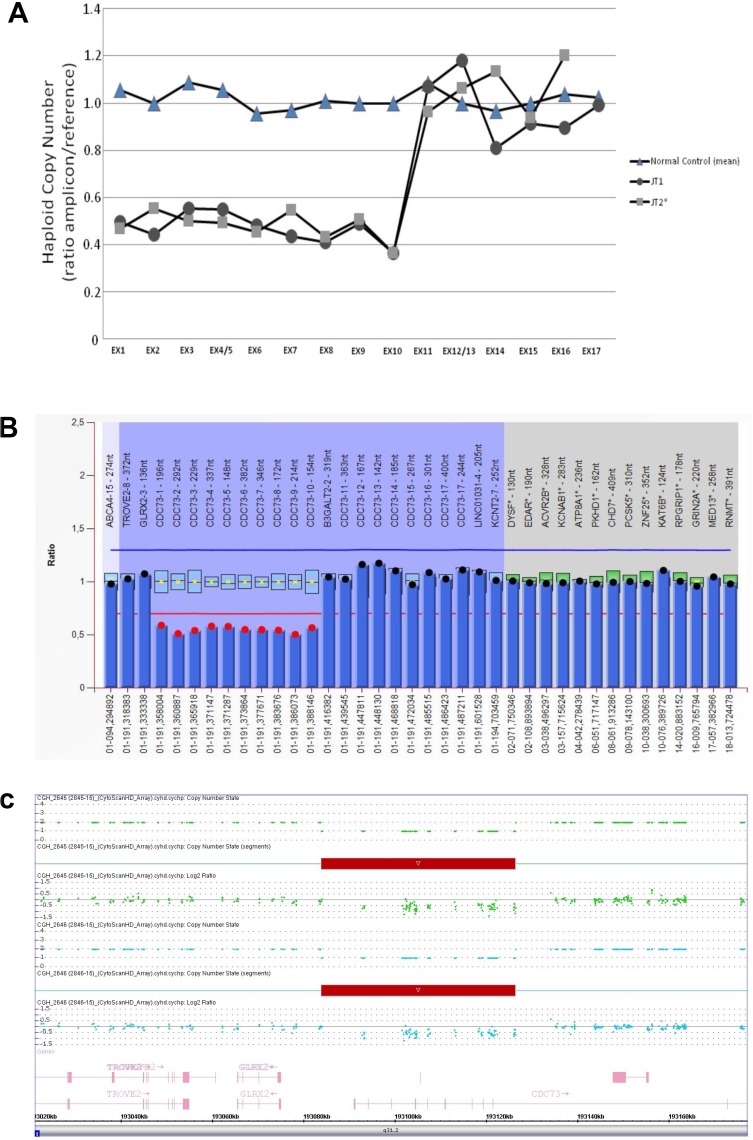
(**A**) Copy number status (± standard error mean) of *CDC73* gene determined by RT-qPCR. Results from normal genomic DNA (2 normal controls mean) and 2 affected members (I:2 and II:6) are visualized by a closed triangle (▲), closed square (■) and a closed circle (●), respectively. Exons 1 to 10 show a hemizigous deletion detectable by the copy number loss of the associated amplicons (see text); (**B**) Bar chart of the MLPA analysis performed on DNA from the proband (II:6) showing the deletion of exons 1–10 of the *CDC73* gene. Deletion is defined with a cut-off ≤ 0,7; (**C**) CytoScan HD Array analysis results of the proband (II:6). Intensity data (log 2 ratio value) of each probe is drawn along chromosome 1 from 193.020 to 193.160 Mb (USCS Genome Browser build February 2009, hg19). The red bar represents the 1q31.2 deletion encompassing exons 1 to 10 of the *CDC73* gene.

Partial deletion of the *CDC73* gene and the hemizygous status in 1 HPT-JT patient (JT2) was detected. The deletion involved the first 10 exons and was confirmed by MLPA and aCGH methodology with the following breakpoints: left breakpoint 193,083,733 –193,083,949 and the right breakpoint 193,126,404–193,126,441 (Figure 1A–1C, respectively). The analysis was then extended to the whole family and the deletion confirmed on all the living affected members and in some unaffected carriers.

### Clinical picture of the HPT-JT family with large deletion

**Figure 2:**
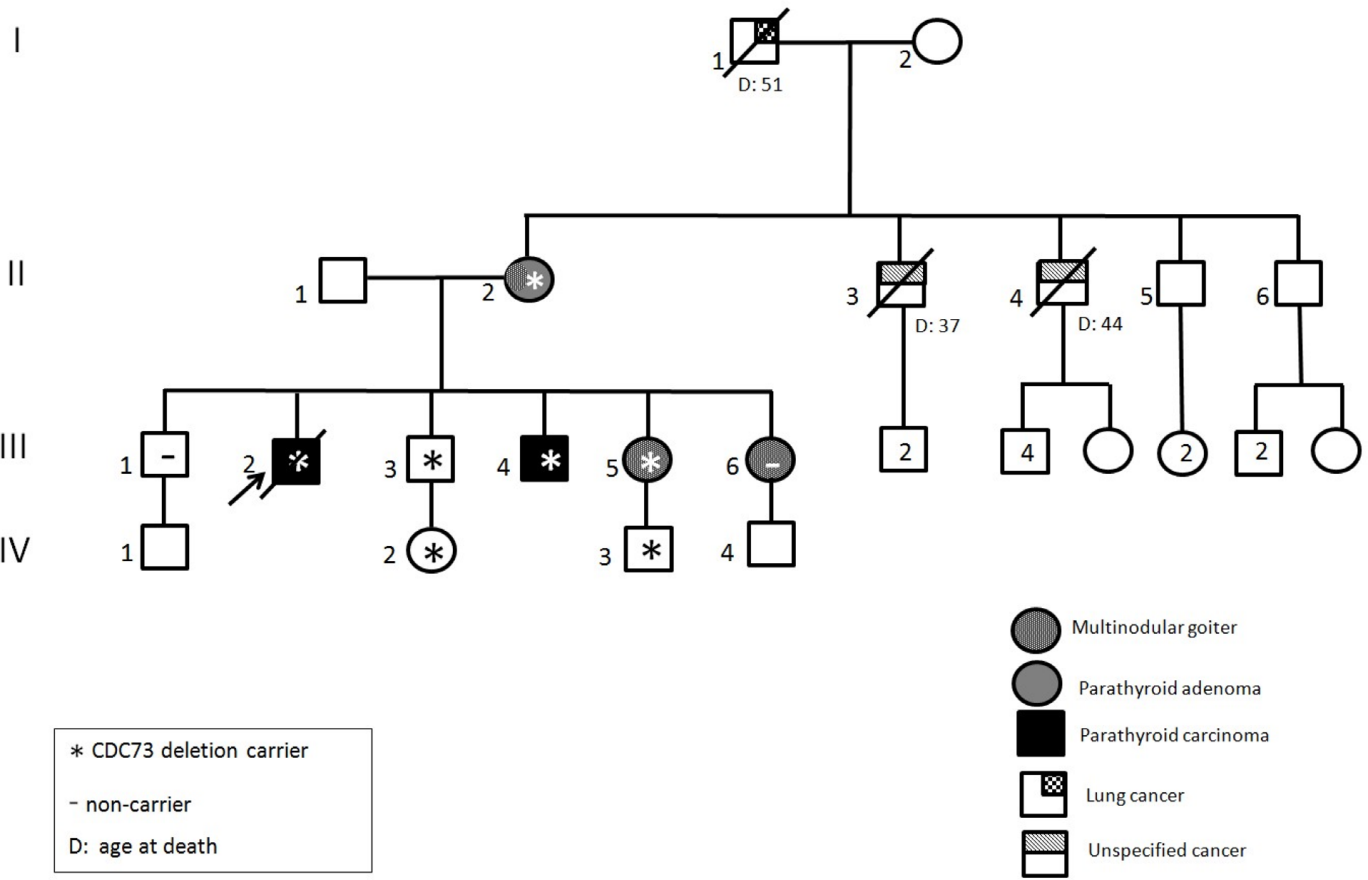
Pedigree of the family carrying the large genomic *CDC73* deletion Clinical status is indicated by open symbols (unaffected) and filled symbols (affected). Filled quadrants indicate a diagnosis as indicated in the inset legend. Proband is indicated by the arrow. The presence/absence (±) of the mutation in tested members is shown. Histological diagnosis of carcinoma is indicated (^#^).

The index case (Figure 2, II:6, 37 ys male) was referred by the Unit of Endocrinology to the Medical Genetics Unit of Sant’Orsola Malpighi Hospital for a previous familial history of PC and adenoma. The eldest brother (II:3), affected by hypercalcemia and nephrocalcinosis, underwent to resection of a fibroma of the maxilla in 1986 at 22 ys, then to a parathyroid adenoma and hyperplasia at 33 ys and finally to an ossifying PC with infiltration of the capsula and vascular invasion when he was 42. He deceased at 46 ys for the consequences of a pathologic femur fracture and presented diffuse metastases at the liver, pelvi and vertebrae. The mother (I:2) had undergone hysterectomy due to uterine leyomiomata at 38 ys and for a parathyroid adenoma at 62 ys. Biochemical assessment of the index case confirmed the diagnosis of hyperparathyroidism with severe hypercalcemia (serum Ca_alb adj_ = 14.9 mg/dL, nr = 8.4–10.2 mg/dL; PTH = 703 pg/mL, nr = 15–65 pg/mL). Imaging investigation (i.e. ultrasonography and Nuclear Magnetic Resonance of the neck) showed an extrathyroidal lesion (max diameter 2 cm) close to the left lobe that, after the surgery, histologically consisted of an ossifying PC with capsular infiltration and invasion of the vessels and of the surrounding tissues. Familial anamnesis also reported the niece (III:2) operated on for uterine fibromas in 2012, at 23 ys. Other relatives did not present any symptoms correlated to the HPT-JT syndrome, while unrelated thyroid disease (multinodular goitre or autoimmune thyroiditis) was shared by almost all the subjects (Figure 2 and Table 1).

**Table 1:** Clinical and biochemical data of the subjects of the HPT-JT family carrying the large deletion

Subjects	Sex	Age at gene testing	HPT-JT Tumour (AaO)	Serum Ca (age)	PTH (age)	Other manifestations (age)
I:2	F	68	PA (62)	9.3 mg/dL (62) 8.8 mg/dL (70)	102 pg/mL (62) 23 pg/mL (70)	uterine fibroids with histerectomy (38) multinodular goiter with left emithyroidectomy (45), non AT multinodular goitre with right emithyroidectomy (62), non AT rheumatic poliamialgia (64) no osteoporosis, no renal lithiasis
II:3	M	NP	fibroma of the maxilla (22) PA + hyperplasia (33) ossifying PC (42) rib metastases from PC (46)	16.8 mg/dL (42)	1358 pg/mL	hypercalcemia associated with nephrocalcinosis: parathyroidectomy left superior and inferior (33) hospitalization for renal failure, nephrocalcinosis, hypercalcemia (42) deceased at age 46 (diffuse metastases)
II:4	M	46	parathyroid hyperplasia (48) ossifying adenoma (48)	11.4 mg/dL (47)	90 pg/mL (47)	micronephrolithiasis (47); multinodular goitre (47) AT (47) no osteoporosis total thyroidectomy amd parathyroidectomy
II:6	M	44	PC (37)	14.9 mg/dL (37)	703 pg/mL (37)	chronic diarrhea (since pediatric age) nephrolithiasis (33) multinodular goitre with follicular lesion at left lobe, total thyroidectomy (37)
II:8	F	42	/	9.3 mg/dL (42)	20 pg/mL (42)	multinodular goitre (21) right thyroidectomy (27) no hypercalcemia, no parathyroid lesion
III:2	F	25	/	9.4 mg/dL (25)	33 pg/mL (25)	uterine fibroids (23)
III:3	M	12	/	9.7 mg/dL (14)	26 pg/mL (14)	/

PA = Parathyroid Adenoma; PC = Parathyroid Carcinoma; AT = Autoimmune Thyroiditis; NA = Not Available; serum Ca normal range = 8.4–10.2 mg/dL; PTH normal range 15–65 pg/mL; NP = Not Performed.

### Parafibromin expression

**Figure 3:**
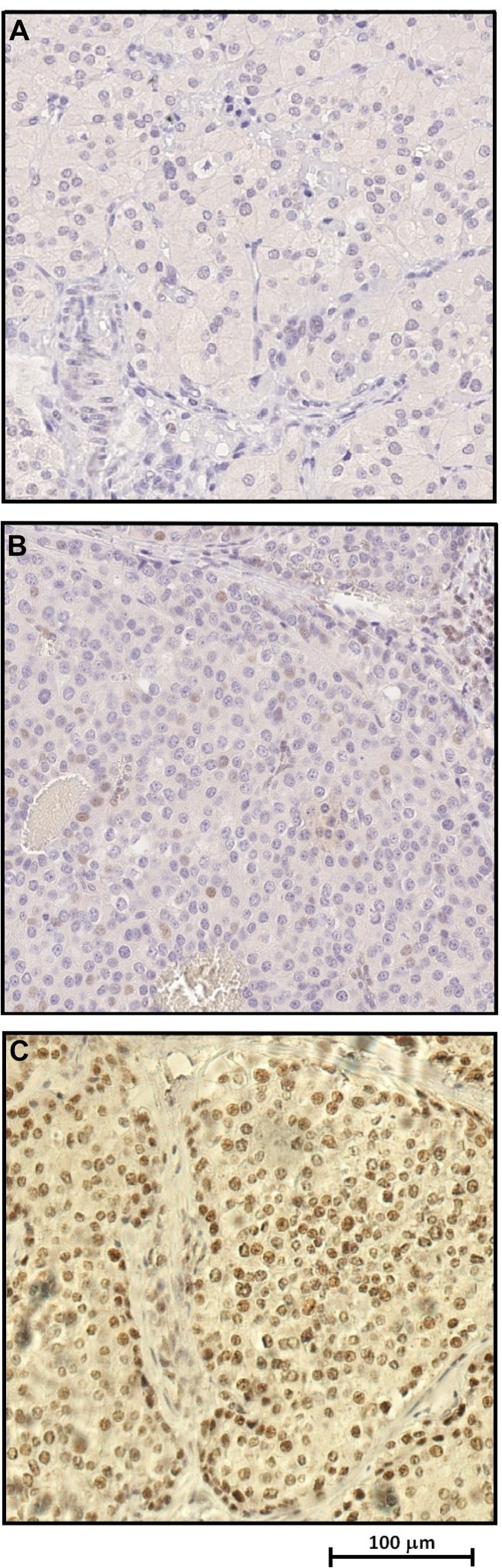
Immunohistochemical analysis revealing loss or significant reduction of parafibromin nuclear expression respectively in parathyroid carcinoma tissue samples (**A**–**B**) compared to unaffected parathyroid tissue (**C**).

Immunoistochemical (IHC) analysis showed complete loss, or considerable reduction, respectively, of parafibromin nuclear expression on parathyroid tumour tissues belonging to two affected patients (II:3 and II:4) compared to the normal tissue (Figure 3A–3C, respectively).

### Analysis of all retrieved published data

References of the papers collected, along with the data for the analysis are in Supplementary Table 1. As shown in Table 2, subjects with a diagnosis of PC carrying a *CDC73* gene mutation (PC^*CDC73*+^) had a significantly lower AaO mean than PC controls affected by the same type of tumor but negative at the molecular screening (PC^*CDC73*–^): 39.15 ± 14.68 vs. 53.00 ± 15.66 years (*p =* 0.017), respectively, as well as for subjects with AA carrying (AA^*CDC73*+^) or not (AA^*CDC73*–^) a *CDC73* gene mutation: 28.00 ± 18.46 vs. 53.86 ± 19.02 years, (*p =* 0.005), respectively. Moreover, among the 57 PC^*CDC73*+^ subjects, AaO mean was significantly lower in subjects with germline (or large genomic deletion) mutations with respect to those with a somatic mutation: 33.95 ± 12.75 vs. 49.83 ± 12.65 years (*p <* 0.001), respectively. Conversely, PC^*CDC73*+^ subjects had a significantly higher Ca^2+^ median levels than controls PC^*CDC73*–^: 15.02 (IQR: 13.10–16.52) vs. 12.72 (IQR: 12.00–13.64) mg/dL (*p =* 0.009), respectively.

**Table 2:** Characteristics of affected subjects with and without a CDC73 mutation (i.e. cases and controls, respectively), according to clinical or histological diagnosis

		Cases (*N* = 192)			Controls (*N* = 19)		comparisons (*p*–value)^#^	
		PC (*N* = 47)	AA (*N* = 12)	TA (*N* = 133)	PC (*N* = 12)	AA (*N* = 7)	P_PC_	P_AA_
Tumor type – *N* (%)	HPT-JT	20 (42.55)	2 (16.67)	76 (57.14)	1 (8.33)	0 (0.00)	---	---
	PC sporadic	19 (40.43)	0 (0.00)	0 (0.00)	11 (91.67)	0 (0.00)		
	PHPT sporadic	0 (0.00)	3 (25.00)	9 (6.77)	0 (0.00)	7 (100.00)		
	FIHP	8 (17.02)	7 (58.33)	48 (36.09)	0 (0.00)	0 (0.00)		
AaO (years)	Mean ± SD	38.17 ± 14.71	28.00 ± 18.46	31.33 ± 15.37	53.00 ± 15.66	53.86 ± 19.02	0.013	0.005
Gender – *N* (%)	M/F (Males%)	29/18 (61.70)	7/5 (58.33)	62/71 (46.62)	4/8 (33.33)	3/4 (42.86)	0.125	0.612
Ca^2+^ (mg/dL)	Median (IQR)	15.07 (13.10–16.70)	13.06 (12.05–15.48)	12.10 (11.20–13.84)	12.72 (12.00–13.64)	13.76 (11.84–15.76)	0.007^*^	0.911^*^
PTH	Median (IQR)	987.75 (453–1469)	300 (136–1500)	247.5 (96–699)	595 (298.5–799)	354 (276–542)	0.133^*^	0.983^*^

Abbreviations: PC: sporadic parathyroid carcinoma, AA: atypical adenoma, TA: typical adenoma, HPT-JT: hyperparathyroidism-jaw tumor syndrome, PHPT: primary hyperparathyroidism, FIHP: familial isolated hyperparathyroidism, PTH: parathyroid hormone, AaO: age at onset, SD: standard deviation, IQR: interquartile range (i.e. first-third quartiles); *p*-values from generalized hierarchical linear models (HGLM) which accounted for family clustering; ^*^p-value from HGLM on log transformed values (due to skewed distribution); ^#^pairwise comparisons between subjects with mutation and controls with respect to their tumor type (i.e. PC, AA).

**Figure 4:**
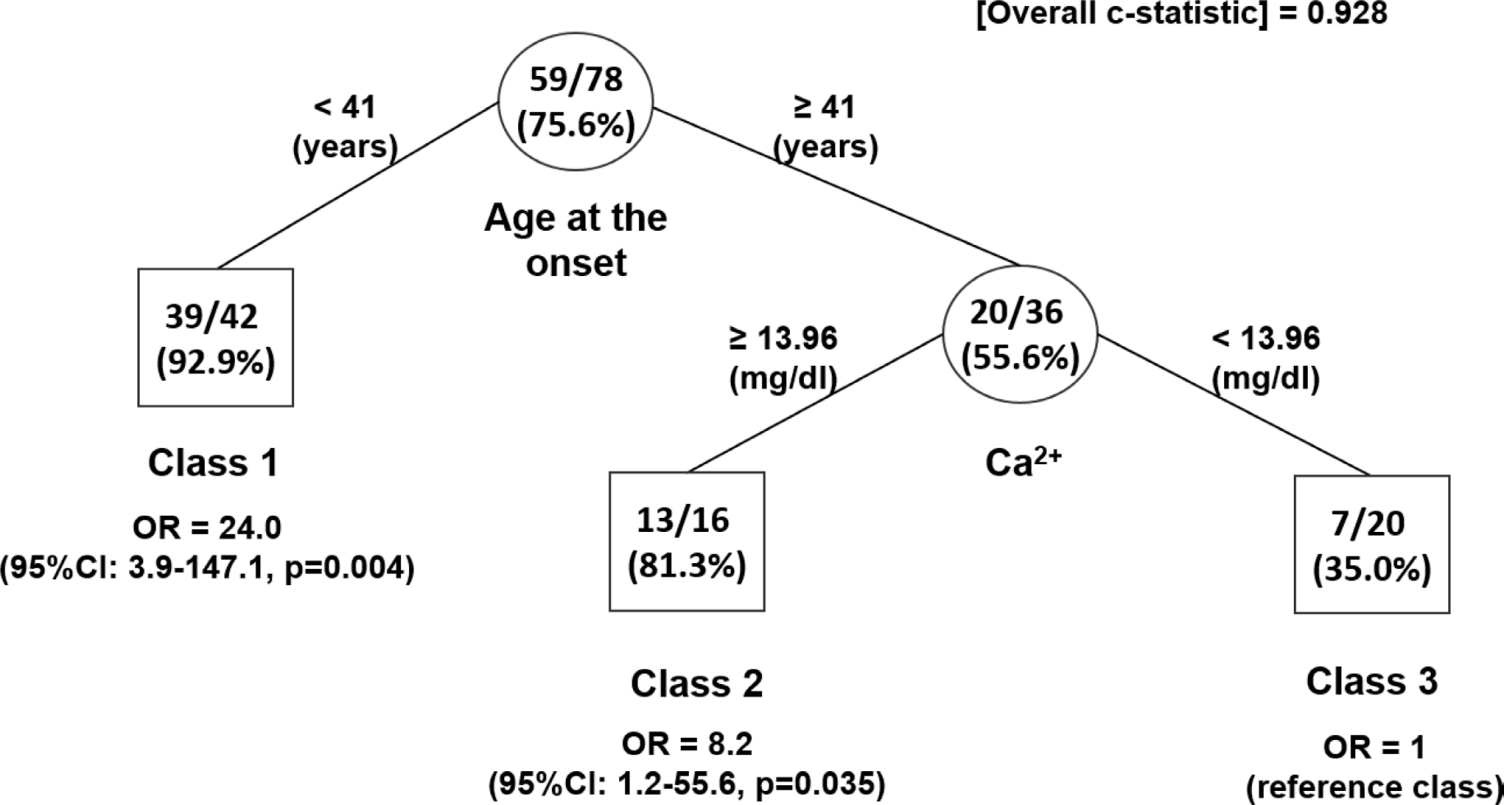
Identification of subgroups at different probability of CDC373 mutation in subjects with diagnosed sporadic parathyroid carcinoma and atypical adenoma only: results of RPART analysis The tree-growing RPART algorithm identified patient subgroups at different probability of *CDC73* mutation. Splitting variables are shown between branches, while condition sending patients to left or right sibling is on relative branch. Class 3 with lowest probability was reference category. Circles indicate subgroups of patients. Squares indicate patient subgroup RPART class. Numbers inside circles and squares represent the number of subjects with *CDC73* mutation (numerator) and the total number of subjects (denominator) and the estimated probability of having the CDC373 mutation (percentage in brackets), respectively. Odds ratios (OR) along with 95% CI were estimated from a HGLM model, which accounted for family clustering.

The tree-based RPART analysis was performed aiming at identifying cut-off points of AaO, Ca^2+^ and PTH levels (candidate splitting variables) to discriminate subjects at different likelihood of having a *CDC73* mutation (Figure 4). A total of three subgroups of patients were identified and patients were finally grouped in respect to AaO and Ca^2+^ levels only. Among the 88 subjects with PC and AA diagnosis, one subject had missing data for all candidate splitting variables (the M1 metastasized parathyroid carcinoma in the Supplementary Table 1) and hence was excluded from the RPART analysis. Therefore, the final sample consisted of 87 subjects. The terminal node with the lowest percentage of subjects with *CDC73* mutation (i.e. the reference Class 3) was represented by the subgroup with AaO ≥ 41.5 years and Ca^2+^ < 13.96 mg/dL. Such terminal node comprised a total of 20 subjects, of whom only 7 of 20 (35.0%) had a *CDC73* mutation. As compared to this subgroup, the Class 1 (which comprised 47 subjects with AaO < 41.5 years) represented the class with the highest probability of having a *CDC73* mutation: indeed 44 out of 47 subjects had a *CDC73* mutation (93.6%; OR = 27.1, 95% CI: 4.3–172.0, *p =* 0.004). A moderate percentage of subjects with *CDC73* mutation was represented by the remaining Class 2, consisting of a subgroup of 20 subjects with AaO ≥ 41.5 years and Ca^2+^ ≥ 13.96 mg/dL: 17 of these 20 subjects (85.0%) carried a *CDC73* mutation (OR = 10.6, 95% CI: 1.6–72.4, *p =* 0.022). As PTH was not included as a splitting variable into the final tree, checking of PTH missing data step was ignored.

## DISCUSSION

Up to date the frequency of large genomic deletions of the *CDC73* locus was never investigated in selected cohorts of sporadic PC and atypical adenoma, resulted negative for coding mutations. Through two different methods (i.e. *ad hoc* RTqPCR and commercial MLPA), we identified 1 large deletion in an Italian HPT-JT family, confirmed by the SNP array. The deletion involved the first 10 exons and was shared by affected but also unaffected subjects, suggesting high heterogeneity of the penetrance, yet reported in other cases [14–15]. IHC showed the loss of parafibromin expression in the two available tumour tissues and although in absence of any constitutional DNA of the subject II:3, the clinical history and the IHC result are consistent with the presence of the large deletion also in this subject. With regard to the sporadic PCs, our negative findings are not unexpected, since they are in line with the frequency elsewhere reported [16, 21]. For these cases, we cannot exclude that *CDC73* genetic lesion might be somatic (coding mutation or LOH, at least in the samples not screened at somatic level) but also that other different genetic determinants may play a role in the onset of the disease. Deep sequencing would undoubtedly help to elucidate this issue as recently reported [21].

**Figure 5:**
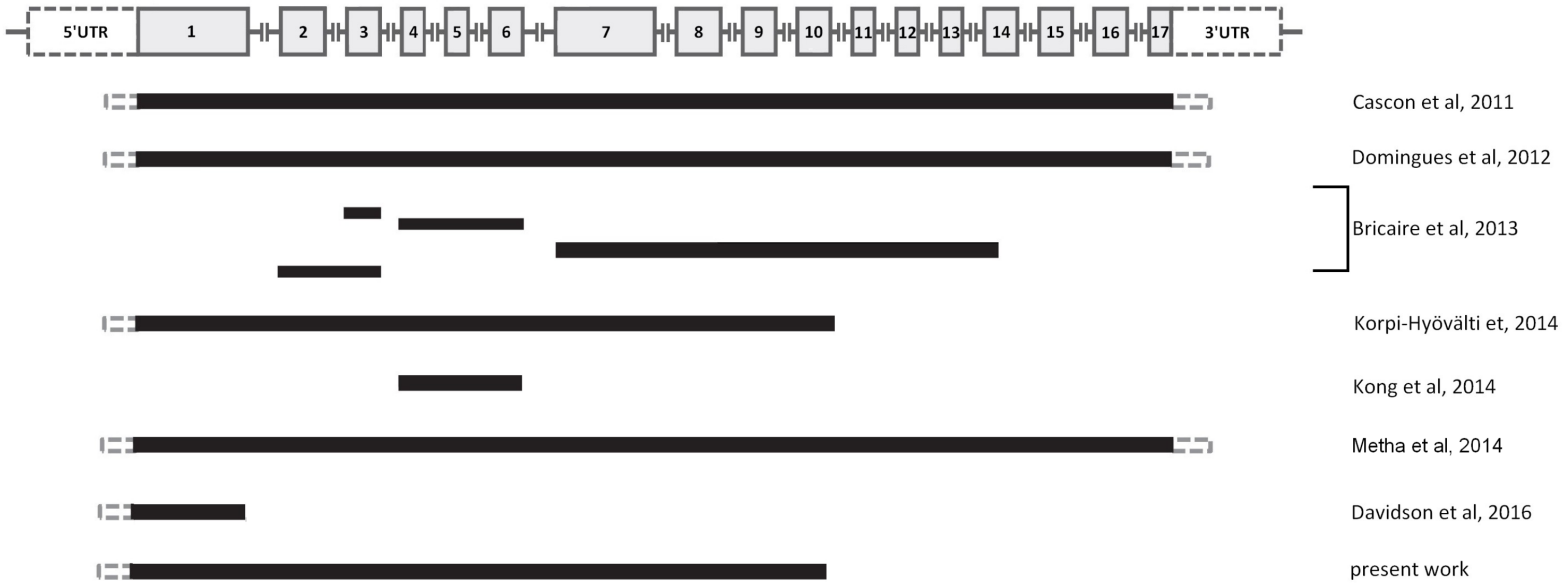
Schematic picture showing the different 11 large genomic deletions at the *CDC73* locus (1q31.2) identified up to the end of the last year, including the novel finding reported in the text (see Supplementary Table 1 for details) On the top, the *CDC73* genomic structure: gray boxes indicate the exons, gray lines indicate the introns (not in scale).

Including the finding here reported, up to the time of preparation of the database we used for statistical analysis (end of the last year), 11 *CDC73* large deletions had been identified in HPT-JT, FHIP or in sporadic PC cases (Figure 5) [14–19]. In one of these studies it was suggested that early onset and severe hypercalcemia (> 3 mMol) may be predictive factors for a possible *CDC73* genetic lesion, independently from the clinical diagnosis (HPT-JT or FHIP or PC) [16].

We searched for a further confirmation of this hypothesis with the aim to possibly better define the cut-off of the predictive biochemical parameters. Thus we conducted an online search for all the papers reporting up to now (last release: June 2016) a *CDC73* mutation and we systematically annotated specific data (AaO, Ca^2+^ and PTH levels, *CDC73* genetic lesion and clinical diagnosis) either for the probands and corresponding relatives (in case of a familial form) (Supplementary Tables 1, 2).

Statistical analysis performed in the pooled dataset revealed different results.

First of all, the tree-structure produced by RPART analysis nicely showed that AaO could be considered the most important predictive variable which best discriminate *per se* the likelihood of having a *CDC73* lesion.

As shown in Figure 4, whenever AaO assumes values < 41.5 years, it achieved the highest discriminatory ability, independently from the levels of Ca^2+^ as it was able to discriminate subjects with the highest likelihood of having a *CDC73* lesion (OR = 27.1, 95% CI: 4.3–172.0, *p* = 0.004 in respect to the reference class). On the other hand, among subjects with AaO ≥ 41.5 ys, the levels of Ca^2+^ represented the secondary important predictive variable as it plays a determinant role in the splitting process (at this step only), because it would be able to discriminate subjects with a higher likelihood of having a *CDC73* lesion (i.e. Ca^2+^ ≥ 13.96 mg/dL, OR = 10.6, 95% CI: 1.6–72.4, *p =* 0.022) compared to those with the lowest likelihood of having such a *CDC73* lesion (i.e. Ca^2+^< 13.96 mg/dl i.e. the reference class).

This work has a limitation: the comparison group we used is affected by internal selection bias. However, it has to be noted that the control group for such a type of analysis had to fulfill specific criteria: available data about AaO, Ca^2+^ and PTH levels for PC/AA resulted negative for the whole molecular screening (germline, somatic and large deletion) of the *CDC73* gene. However, to our knowledge, such kind of data are not available in literature, apart from the ones we reported here. A further confirmation of our observation will be desirable once other cohorts will be available.

Moreover, other additional features could be involved in this analysis as the histology or the expression of the parafibromin protein on tumour tissues [21]. A recent retrospective study on prognostic/diagnostic criteria of PC in the Finnish population [22], used a similar approach: the Authors included also the histological features of the 32 PC, 28 AA and 72 TA to better refine the cut-offs of their parameters. In our study, we did not include the histology data for two reasons: first of all, not all the papers collected reported such data. Second and more important: our approach is based on data that are usually available only in first diagnosis, being the main aim, to possibly identify biomarkers for a presurgical diagnosis of malignancy. Instead, before the surgery, the suspect of malignancy could drive towards the better surgery option impacting on disease local recurrence, long term overall survival, proper follow up of the patients [4]. Today, only the presence of metastasis can provide unequivocal evidence of malignity. However, as clearly emphasized by the data collected from literature and here reported, on a total of 57 (histologically diagnosed) PCs, metastases were reported only in 18 (31.5%, Supplementary Table 1), and almost all identified up to 6–10 years after the first surgery.

With regard to the previous study, it is very important to note that, despite the different set of data, our results are in line with the previous one.

Although we are aware of the limitations of our study, we believe the strength and novelty of our work lies in the comprehensive systematic analysis of data retrieved from the scientific literature that, to our knowledge, has never been reported. Instead, based on clinical experiences, we attempted to define, what can be the most reliable biochemical cut-offs of severe hypercalcemia and age at onset that had been only generally associated to malignant parathyroid lesions in the last decades. We are confident that it may help in the identification of subjects at risk to have a *CDC73* gene mutation and consequently at risk to develop an aggressive parathyroid disease on the basis of a patient’s blood sample test.

In conclusion, we report a novel large *CDC73* deletion in an Italian HPT-JT Family and we would stress the importance to implement a complete screening protocol in subjects with suspected CDC73 associated disorders (HPT-JT or sporadic PC). We suggest as first step: i) direct sequencing of all the 17 exons of the CDC73 coding region and, if the screening had a negative result, as second analysis level: ii) the search for large genomic deletions at the CDC73 locus (chr 1q31.2) by commercial MLPA kit or by classic RTqPCR protocol. Moreover our analysis on 202 different affected subjects carrying for a *CDC73* gene mutation alerts that early AaO < 41.5 ys and Ca^2+^ > 3.49 mMol are associated with a high risk of a *CDC73* genetic lesion.

## MATERIALS AND METHODS

### Clinical cases

Our Local Ethic Committee at the IRCCS Casa Sollievo della Sofferenza Hospital in San Giovanni Rotondo (FG), approved the protocol and informed consent was obtained from all the participants. Twenty patients with a clinical or histological diagnosis of: sporadic parathyroid carcinoma (PC = 11), atypical adenoma (AA = 7) or Hyperparathyroidism with Jaw Tumour (HPT-JT = 2) syndrome were recruited. This survey included 3 PC, 4 AA and 1 HPT-JT, previously reported as negative at the molecular screening [20], but not analyzed for genomic deletion at the corresponding *CDC73* locus.

### Genetic screening

Genomic DNA was extracted from peripheral blood of all the affected and unaffected subjects, using standard methods. The whole coding sequence of the *CDC73* gene including the exon-intron boundaries was by PCR amplified and direct sequencing of all the 17 exons (16 amplicons) was performed as previously described [20]. LOH was carried out as described [20] on a limited number of samples (nr 12: 2 JT, 4 PC, 6 AA) due to the unavailability of some DNA tumour tissues.

### RTqPCR

A new protocol was developed to provide a sensitive method for detecting large deletions encompassing the *CDC73* gene and the 1q32.1 locus. All primers were designed (Primer Express 2.0 software, Applied Biosystems) and tested for specificity (BLAT software at http://genome.ucsc.edu/cgi-bin/hgBlat). Primer sets identify 15 amplicons covering all the *CDC73* gene exons (exons 1–17). Analysis was carried out on the 384-well ABI Prism 7900 Sequence Detection System through the measurements of the amplicons Copy Number (CN) using a relative approach of quantification with SYBR-Green I. Calculation of the gene copy number was made using the 2-deltadeltaCT method. Outlier values with a difference between Ct and Ct mean > 0.3 were excluded from further data analysis. For normalization of the relative amount, the gene copy numbers of MOX2 (3q13.2) were used. By using this method, a Diploid Copy Number (D-CN) of 1.0 ± 0.2 is expected for a normal sample and a value of 0.5 ± 0.2 for a sample with *CDC73* deletion (Haploid Copy Number, H-CN).

### MLPA

Recently, the commercial kit based on the Multiple Ligation Probe Assay (MLPA) for search of genomic deletion at the *CDC73* locus was developed: the P466 kit (MRC Holland) was applied following the manufacturer’s instructions.

### SNP array

Whole-genome copy number variation (CNV) analysis was carried out with the CytoScan HD array platform (Affymetrix) on leukocyte DNA of the subjects (I:2, II:1, II:4, II:6, II:8, II:10, III:2 and III:3). The array contains more than 2.6 million markers for copy number analysis and approximately 750,000 SNPs that fully genotype with greater than 99% accuracy. The CytoScanHD assay was performed according to the manufacturer’s protocol, starting with 250 ng DNA. Hybridization to the microarray was carried out in a specific Oven 645 while subsequent washing and staining were performed using the Fluidics Station 450. The array was then scanned with the Scanner 3000 7G and both the quality control step and copy number analysis were performed using Chromosome Analysis Suite Software version 2.0. The raw data file (.CEL) was normalized using the default options and an unpaired analysis was performed using as baseline 270 HapMap samples to obtain the copy number value from .CEL files while the amplified and/or deleted regions were detected using a standard Hidden Markov Model (HMM) method. Base pair positions were obtained from the University of California Santa Cruz (UCSC) Genome Browser (http://genome.ucsc.edu/cgi-bin/hgGateway), build GRCh37 (hg19).

### Immunohistochemistry

Representative 4 micron-thick cell block sections of formalin-fixed paraffin embedded (FFPE) parathyroid tumor tissues were rehydrated in pH 7.5 buffer and processed for standard staining. Antigen retrieval was performed heating the slides in 0.01 M citrate buffer (pH 6.0) in a bath for 20 min at 97° C. After blocking endogenous peroxidase activity in 0.3% hydrogen peroxide and methanol solution for 15 min, tissue sections were stained respectively with a primary monoclonal anti-parafibromin antibody (1:200 dilution; clone 2H1, sc33638, both from Santa Cruz Biotechnology, Santa Cruz, CA) for 20 min.

Slides were then incubated with a commercially available detection kit (EnVision™ FLEX+, Dako, Glostrup, Denmark) following the manufacturer’s instructions, developing peroxidase activity with 3–3′-diaminobenzidine. Finally, slides were counterstained with hematoxylin, dehydrated and mounted. The specificity of all reactions was checked by replacing the primary antibody with an unrelated mouse immunoglobulin at a comparable dilution or using normal serum alone. Positive and negative controls were used as appropriate.

### MEDLINE search strategy (data extraction)

A systematic search for the full articles reporting data concerning: *CDC73* mutation, clinical and histological diagnosis, AaO of the disease, gender, Ca^2+^ and PTH levels was conducted through the public available Medline database (https://www.ncbi.nlm.nih.gov/pubmed; last access: June 2016) using as keywords: “*CDC73*”, “HRPT2”, “parafibromin”, “parathyroid carcinoma”, “hyperparathyroidism with jaw tumour”, “HPT-JT”. Full texts were examined by one/more author (LC and VG), who assessed their eligibility and extracted data. A total of 77 different full articles and 1 not published report were identified: 25 articles were excluded because of some missed data information; 2 papers in Chinese were excluded because data cannot be retrieved at the time of this report. The 51 remaining reports described a total of 197 patients with a *CDC73* gene mutation corresponding to 76 single occurrences (66 coding + 10 large deletions). Including the 7 members of HPT-JT family shown below and carrying a *CDC73* large deletion, we get a total of 204 patients mutated by 77 single occurrences of which 11 were large deletions (Supplementary Table 1 and Supplementary References. Correlation between the different clinical parameters (age, Ca2^+^, PTH) in subjects carrying a CDC73 mutation or not, with respect to clinical or histological diagnosis and are in Supplementary Tables 2 and 3).

### Exclusion/inclusion criteria

Patients/relatives were included in the analysis whether at least 3 out of 4 variables (histology, AaO, blood Ca^2+^ and PTH levels) were available. We exceptionally included 2 cases of metastasized parathyroid carcinoma (Supplementary Table 1) that were partially used, since not all the data were available. Moreover, as the LG1 [14] was also referenced in a different paper [16], but it was not possible to deduce the kinship between the two probands, for this family/large deletion we considered both. All the data collected, normalized as needed, were summarized with regard to the type of *CDC73* mutation (germline, somatic, large deletion) and the clinical diagnosis (HPT-JT, PC, FHIP or PHPT). For familial cases, same data were collected along with the degree of kinship (with reference to the proband). The 12 cases of sporadic PC and 7 AA resulted negative in the present screening (either for coding mutations and large deletions) were included as comparison control group. Finally we were able to define 202 subjects with a *CDC73* genetic lesion as “cases” whereas the 19 subjects without any *CDC73* genetic lesion as “controls”.

### Statistical methods

Data were reported as mean ± standard deviation (SD) or frequency and percentages for continuous and categorical variables, respectively. For each continuous variable, the assumption of normality distribution was checked by means of Q-Q plots and Shapiro-Wilks test. For skewed continuous variables, medians along with interquartile range (IQR) (i.e. first-third quartiles) were reported instead of means.

Comparisons between cases and controls were assessed using hierarchical generalized linear models (HGLM) because the comparisons were performed with regard to continuous variables (i.e. AaO, Ca^2+^ and PTH levels) and a categorical variable (i.e. gender). Such class of models accounted for “family clustering” (i.e. observations are not independent as they were collected within families see also the family size distribution in Supplementary Table 4), where normal and binomial distributions of the error term were assumed to model continuous and categorical variables, respectively. Comparisons between Ca^2+^ and PTH levels were assessed on logarithm transformed scale, due to the skewed distribution of their original values (i.e. log-Ca^2+^ and log-PTH, respectively, see also Supplementary Figure 1). Pairwise comparisons between cases and controls were performed, in respect to PC and AA diagnosis only (because there were no TA controls). In details, separate analysis of variance models (which also accounted for the hierarchy structure) were performed for each interested variable at issue (i.e. AaO, log-Ca^2+^, log-PTH levels and gender), including: the presence of the *CDC73* mutation (i.e. being a case or control), the type of clinical or histological diagnosis (i.e. PC or AA diagnoses) and mutation-by-diagnosis interaction term. To assess pairwise comparisons, suitable statistical contrasts of least square means were therefore estimated within each HGLM. Moreover, comparisons of clinical characteristics (i.e. AaO, Ca^2+^ and PTH levels) between type of mutation (i.e. germline/large genomic deletion vs. somatic mutations) were performed among cases only.

Recursive PArtitioning and Regression survival Tree (RPART) [23, 24] algorithm was performed among PC and AA cases (*N* = 69) and PC and AA controls (*N* = 19) in order to predict the likelihood of having a *CDC73* mutation (i.e. the probability to be a case) on the basis of observed baseline clinical variables (i.e. AaO, Ca^2+^ and PTH levels). This tree-based method recursively splits the data in subgroups, choosing the best binary split for each considered variable at issue, to identify the most homogeneous sets (in terms of proportion of subjects with *CDC73* mutations) which also were the most heterogeneous with regard to one other, until the following user-defined conditions (stopping rules) are met: subgroups either reach a minimum size of 10 subjects (i.e. terminal node) or no improvement can be made (i.e. in terms of a complexity parameter which should minimize the cross-validated error rate). A split can be performed only if the splitting variable was observed in all subjects within the node (i.e. no missing values). In case the splitting variable was missing for some subjects, the best “surrogate” split was detected (among all candidate splitting variables, with the exclusion of the one involved for the original split) and used for these subjects. To obtain more robust and stable splits, a 10-fold cross-validation at each splitting node was adopted. The terminal node with the lowest probability of having a *CDC73* mutation was represented as the reference class. Odds ratios (OR) contrasting in respect to reference class were eventually estimated by a HGLM model with random intercept only, along with 95% confidence interval (95% CI).

A two-sided *p*-value < 0.05 was considered for statistical significance. All statistical analyses were performed using SAS Release 9.4 (SAS Institute, Cary, NC, USA) and R (CRAN) Comprehensive R Archive Network (CRAN), version 3.2.

## SUPPLEMENTARY MATERIALS FIGURE AND TABLES




