# Supplementary material for: Large deletion at the CDC73 gene locus and search for predictive markers of the presence of a CDC73 genetic lesion
Source: Oncotarget. 2018 Apr 17;9(29):20721–33. doi: 10.18632/oncotarget.25067 (PMC5945533; doi:10.18632/oncotarget.25067)
Supplement: Supplementary file 1 [file oncotarget-09-20721-s001.pdf]

Large deletion at the *CDC73* gene locus and search for predictive markers of the presence of a *CDC73* genetic lesion

SUPPLEMENTARY MATERIALS

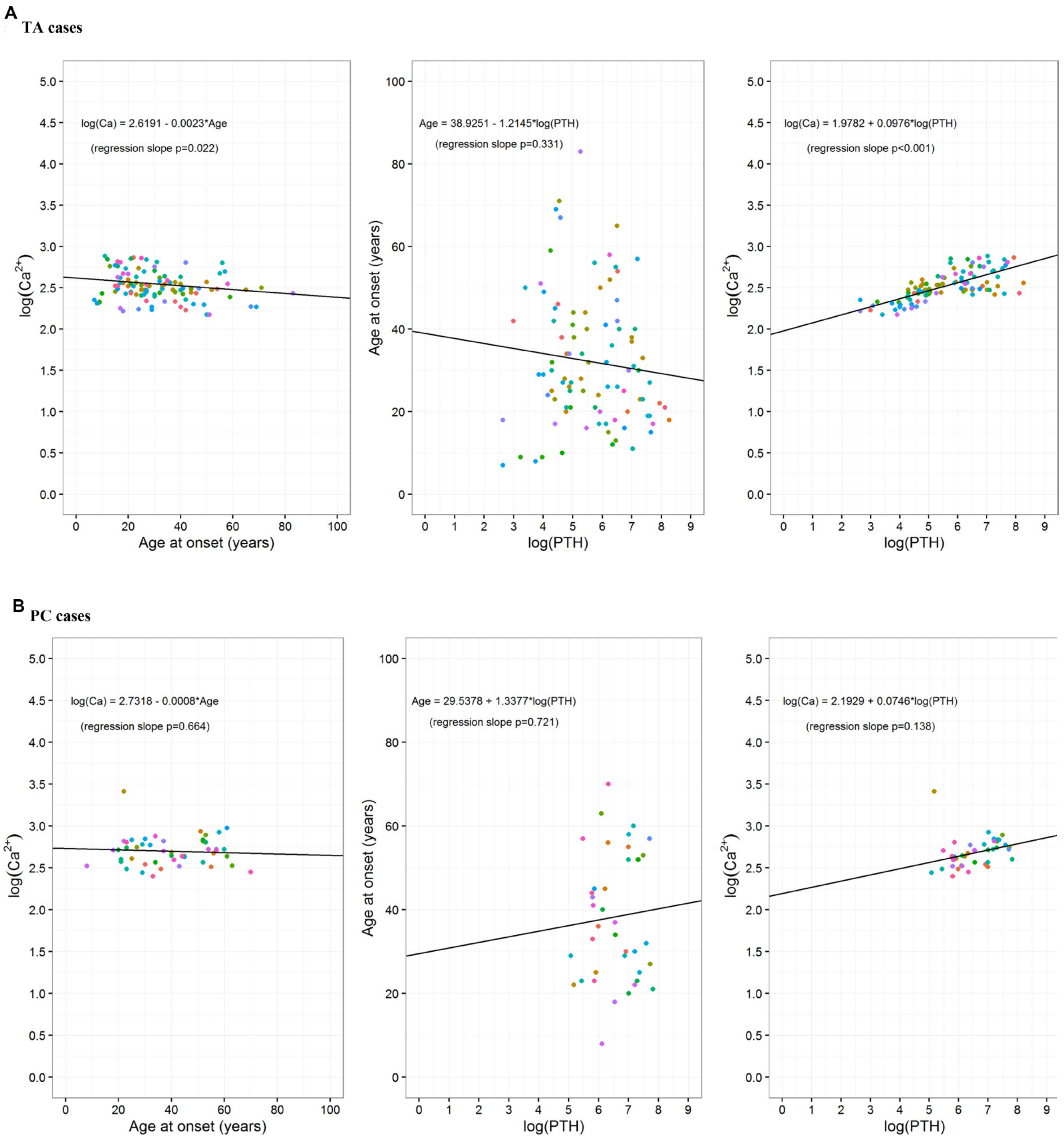

C
AA cases

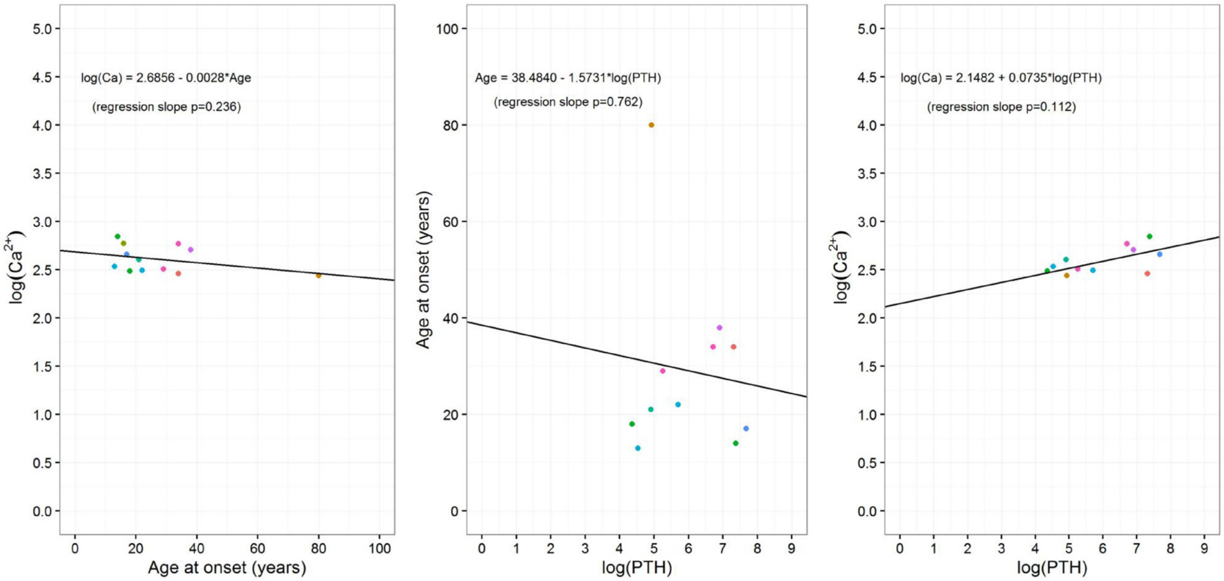

D
PC controls

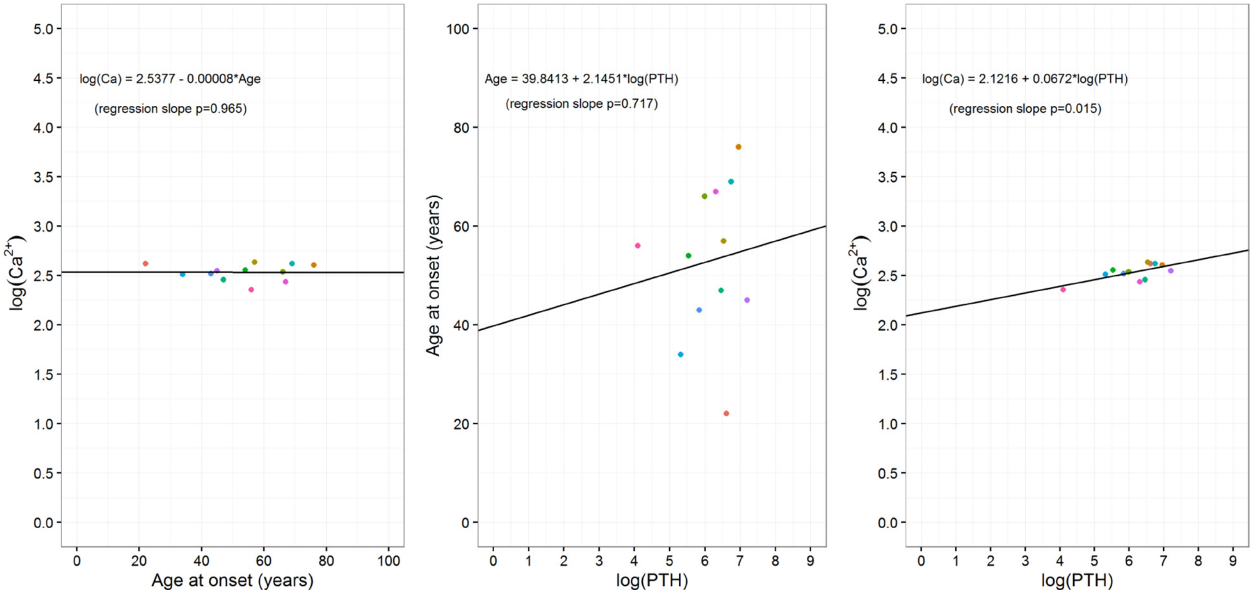

E
AA controls

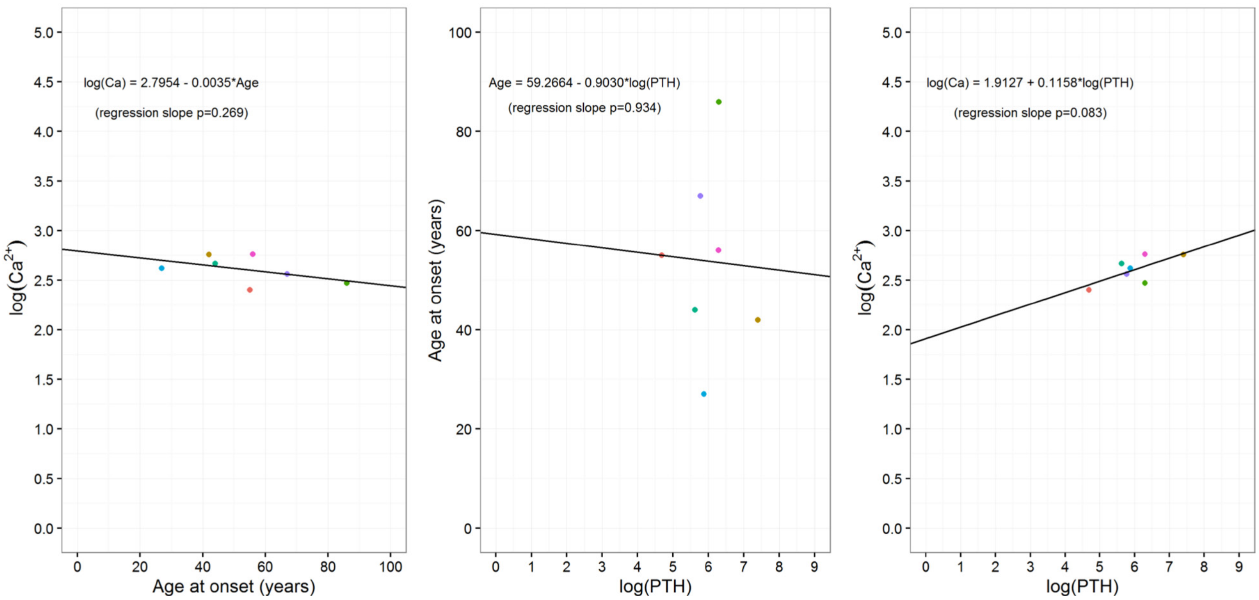

Supplementary Figure 1: Scatterplots of Ca^{2+} levels vs. age at the onset vs. PTH levels in cases (panels: **A**, **B**, **C**) and controls (panels: **D**, **E**) with respect to clinical diagnosis (i.e. PC: sporadic parathyroid carcinoma, AA: atypical adenoma, TA: typical adenoma), separately. Regression lines were fitted from hierarchical linear models which account for family clustering. Points with different colors are referred to different families and/or sporadic cases.

Supplementary Table 1: List of the papers used for statistical analysis. See Supplementary_Table_1

Supplementary Table 2: Estimated regression slopes, coefficient of determination (R^2) and the corresponding correlation coefficient between age at onset (AaO), Ca^{2+} and PTH levels (in logarithm scale) in subjects with a CDC73 mutation (cases) and in those without a CDC73 mutation (controls), according to the different clinical or histological diagnosis (separate analysis for each subgroup)

Group	Clinical diagnosis	*N*[*]	Variables		Slope (SE)[§]	*p*-value for slope[§]	R^2 [#]	Corr (square root R^2)[#]
			Y	X				
Cases (*N* = 202)	PC	49	°Ca^{2+}	AaO	−0.0008 (0.0016)	0.664	0.0691	−0.263
		40	°Ca^{2+}	°PTH	0.0746 (0.0311)	0.138	0.6389	0.799
		39	AaO	°PTH	1.3377 (3.2513)	0.721	0.1125	0.335
	AA	12	°Ca^{2+}	AaO	−0.0028 (0.0022)	0.236	0.1371	−0.370
		11	°Ca^{2+}	°PTH	0.0735 (0.0269)	0.112	0.5528	0.744
		11	AaO	°PTH	−1.5731 (5.0327)	0.762	0.0107	−0.103
	TA	105	°Ca^{2+}	AaO	−0.0023 (0.0009)	0.022	0.0554	−0.235
		96	°Ca^{2+}	°PTH	0.0976 (0.0100)	<0.001	0.7122	0.844
		95	AaO	°PTH	−1.2145 (1.2417)	0.331	0.0102	−0.101
Controls (*N* = 19)	PC	12	°Ca^{2+}	AaO	−0.0001 (0.0017)	0.965	0.0002	−0.014
		12	°Ca^{2+}	°PTH	0.0672 (0.0229)	0.015	0.4624	0.680
		12	AaO	°PTH	2.1451 (5.7561)	0.717	0.0137	0.117
	AA	7	°Ca^{2+}	AaO	−0.0035 (0.0028)	0.269	0.2362	−0.486
		7	°Ca^{2+}	°PTH	0.1158 (0.0537)	0.083	0.4816	0.694
		7	AaO	°PTH	−0.9030 (10.3139)	0.934	0.0015	−0.039

Abbreviations: PC: sporadic parathyroid carcinoma, AA: atypical adenoma, TA: typical adenoma. [*]N: Number of subjects without missing values for both investigated variables. [°]Variables expressed in logarithm-scale. [§]Estimates of regression slopes (variable Y regressed on X), along with their standard errors (SE) and their *p*-values. In cases, estimates and *p*-values were derived from hierarchical linear models which accounted for family clustering . In controls, estimates and *p*-values were derived from simple linear regression models. [#]Coefficient of determination calculated assessing the reduction in residual variance attributable the presence of the covariate (X) with respect to the model without X and the corresponding correlation was derived by the square root of R^2 (placing positive or negative sign according to the slope's sign).

Supplementary Table 3: Characteristics of affected subjects with a CDC73 mutation, according to clinical or histological diagnosis and type of mutation (i.e. germline / large genomic deletions vs. somatic)

		Germline/Large deletions	Somatic	*p*-value
PC ($N = 47$)	N (%)	34 (72.34)	13 (27.66)	---
	AaO (years)	33.45 ± 13.34	50.15 ± 10.98	<0.001
	Ca^{2+}(mg/dL)	15.20 (13.10–16.52)	14.50 (13.60–16.70)	0.799*
	PTH	1052.50 (462.14–1469.00)	527.00 (422.00–1294.50)	0.544*
AA ($N = 12$)	N (%)	11 (91.67)	1 (8.33)	---
	AaO (years)	23.27 ± 8.93	80.00	---
	Ca^{2+}(mg/dL)	13.52 (12.10–15.96)	11.48	---
	PTH	561.00 (136.00–1500.00)	139	---
TA ($N = 133$)	N (%)	130 (97.74)	3 (2.26)	---
	AaO (years)	31.26 ± 15.52	34.33 ± 6.51	0.734
	Ca^{2+}(mg/dL)	12.10 (11.20–13.89)	12.40 (11.28–12.72)	0.598*
	PTH	313.00 (94.00–718.00)	121.00 (113.00–149.00)	0.171*

Abbreviations: PC: sporadic parathyroid carcinoma, AA: atypical adenoma, TA: typical adenoma, PTH: parathyroid hormone, AaO: age at onset; *p*-value from generalized hierarchical linear models (HGLM) which accounted for family clustering
**p*-value from HGLM on log transformed values.

Supplementary Table 4: Family size distribution among cases ($N = 192$)

Single members (sporadic cases)	62 (32.29%)
2 members	11 ($N = 22$, 11.46%)
3 members	8 ($N = 24$, 12.50%)
≥4 members	13 ($N = 84$, 43.75%)

REFERENCES

1. Abdulla AG, O'Leary EM, Isorena JP, Diaz MF, Yeh MW. Recurrent hyperparathyroidism and a novel nonsense mutation in a patient with hyperparathyriodism-jaw tumor syndrome. Endocr Pract. 2013; 19:e134–7.
2. Bradley KJ, Cavaco BM, Bowl MR, Harding B, Young A, Thakker RV. Utilisation of a cryptic non-canonical donor splice site of the gene encoding parafibromin is associated with familial isolated primary hyperparathyroidism. J Med Genet. 2005; 42:e51.
3. Bricaire L, Odou MF, Cardot-Bauters C, Delemer B, North MO, Salenave S, Vezzosi D, Kuhn JM, Murat A, Caron P, Sadoul JL, Silve C, Chanson P, et al. Frequent large germline HRPT2 deletions in a French National cohort of patients with primary hyperparathyroidism. J Clin Endocrinol Metab. 2013; 98:E403–8.
4. Carlson AL, Smith CL. Primary hyperparathyroidism and jaw tumor syndrome: a novel mutation of the HRPT2 gene. Endocr Pract. 2008; 14:743–7.
5. Carpten JD, Robbins CM, Villablanca A, Forsberg L, Presciuttini S, Bailey-Wilson J, Simonds WF, Gillanders EM, Kennedy AM, Chen JD, Agarwal SK, Sood R, Jones MP, et al. HRPT2, encoding parafibromin, is mutated in hyperparathyroidism-jaw tumor syndrome. Nat Genet. 2002; 32:676–80.
6. Cascón A, Huarte-Mendicoa CV, Javier Leandro-García L, Letón R, Suela J, Santana A, Costa MB, Comino-Méndez I, Landa I, Sánchez L, Rodríguez-Antona C, Cigudosa JC, Robledo M. Detection of the first gross CDC73 germline deletion in an HPT-JT syndrome family. Genes Chromosomes Cancer. 2011; 50:922–9.
7. Cavaco BM, Guerra L, Bradley KJ, Carvalho D, Harding B, Oliveira A, Santos MA, Sobrinho LG, Thakker RV, Leite V. Hyperparathyroidism-jaw tumor syndrome in Roma families from Portugal is due to a founder mutation of the HRPT2 gene. J Clin Endocrinol Metab. 2004; 89:1747–52.
8. Cavaco BM, Santos R, Félix A, Carvalho D, Lopes JM, Domingues R, Sirgado M, Rei N, Fonseca F, Santos JR, Sobrinho L, Leite V. Identification of de novo germline mutations in the HRPT2 gene in two apparently sporadic cases with challenging parathyroid tumor diagnoses. Endocr Pathol. 2011; 22:44–52.
9. Cetani F, Ambrogini E, Viacava P, Pardi E, Fanelli G, Naccarato AG, Borsari S, Lemmi M, Berti P, Miccoli P, Pinchera A, Marcocci C. Should parafibromin staining replace HRTP2 gene analysis as an additional tool for histologic diagnosis of parathyroid carcinoma? Eur J Endocrinol. 2007; 156:547–54.
10. Cetani F, Banti C, Pardi E, Borsari S, Viacava P, Miccoli P, Torregrossa L, Basolo F, Pelizzo MR, Rugge M, Pennelli G, Gasparri G, Papotti M, et al. CDC73 mutational status and loss of parafibromin in the outcome of parathyroid cancer. Endocr Connect. 2013; 2:186–95.
11. Cetani F, Pardi E, Ambrogini E, Viacava P, Borsari S, Lemmi M, Cianferotti L, Miccoli P, Pinchera A, Arnold A, Marcocci C. Different somatic alterations of the HRPT2 gene in a patient with recurrent sporadic primary hyperparathyroidism carrying an HRPT2 germline mutation. Endocr Relat Cancer. 2007; 14:493–9.
12. Cetani F, Pardi E, Borsari S, Viacava P, Dipollina G, Cianferotti L, Ambrogini E, Gazzerro E, Colussi G, Berti P, Miccoli P, Pinchera A, Marcocci C. Genetic analyses of the HRPT2 gene in primary hyperparathyroidism: germline and somatic mutations in familial and sporadic parathyroid tumors. J Clin Endocrinol Metab. 2004; 89:5583–91.
13. Chiofalo MG, Sparaneo A, Chetta M, Franco R, Baorda F, Cinque L, Granatiero M, D'Agruma L, Pezzullo L, Scillitani A, Guarnieri V. A novel CDC73 gene mutation in an Italian family with hyperparathyroidism-jaw tumour (HPT-JT) syndrome. Cell Oncol (Dordr). 2014; 37:281–8.
14. Davidson JT, Lam CG, McGee RB, Bahrami A, Diaz-Thomas A. Parathyroid Cancer in the Pediatric Patient. J Pediatr Hematol Oncol. 2016; 38:32–7.
15. Domingues R, Tomaz RA, Martins C, Nunes C, Bugalho MJ, Cavaco BM. Identification of the first germline HRPT2 whole-gene deletion in a patient with primary hyperparathyroidism. Clin Endocrinol (Oxf). 2012; 76:33–8.
16. Frank-Raue K, Haag C, Schulze E, Keuser R, Raue F, Dralle H, Lorenz K. CDC73-related hereditary hyperparathyroidism: five new mutations and the clinical spectrum. Eur J Endocrinol. 2011; 165:477–83.
17. Ghemigian A, Ghemigian M, Popescu I, Vija L, Petrova E, Dumitru N, Dumitru I. Familial isolated primary hyperparathyroidism due to HRPT2 mutation. Hormones (Athens). 2013; 12:454–60.
18. Guarnieri V, Battista C, Muscarella LA, Bisceglia M, de Martino D, Baorda F, Maiello E, D'Agruma L, Chiodini I, Clemente C, Minisola S, Romagnoli E, Corbetta S, et al. CDC73 mutations and parafibromin immunohistochemistry in parathyroid tumors: clinical correlations in a single-centre patient cohort. Cell Oncol (Dordr). 2012; 35:411–22.
19. Guarnieri V, Scillitani A, Muscarella LA, Battista C, Bonfitto N, Bisceglia M, Minisola S, Mascia ML, D'Agruma L, Cole DE. Diagnosis of parathyroid tumors in familial isolated hyperparathyroidism with HRPT2 mutation: implications for cancer surveillance. J Clin Endocrinol Metab. 2006; 91:2827–32.
20. Iacobone M, Masi G, Barzon L, Porzionato A, Macchi V, Ciarleglio FA, Palù G, De Caro R, Viel G, Favia G. Hyperparathyroidism-jaw tumor syndrome: a report of three large kindred. Langenbecks Arch Surg. 2009; 394:817–25.
21. Juhlin C, Larsson C, Yakoleva T, Leibiger I, Leibiger B, Alimov A, Weber G, Höög A, Villablanca A. Loss of parafibromin expression in a subset of parathyroid adenomas. Endocr Relat Cancer. 2006; 13:509–23.

22. Juhlin CC, Haglund F, Obara T, Arnold A, Larsson C, Höög A. Absence of nucleolar parafibromin immunoreactivity in subsets of parathyroid malignant tumours. Virchows Arch. 2011; 459:47–53.
23. Juhlin CC, Villablanca A, Sandelin K, Haglund F, Nordenström J, Forsberg L, Bränström R, Obara T, Arnold A, Larsson C, Höög A. Parafibromin immunoreactivity: its use as an additional diagnostic marker for parathyroid tumor classification. Endocr Relat Cancer. 2007; 14:501–12.
24. Kelly TG, Shattuck TM, Reyes-Mugica M, Stewart AF, Simonds WF, Udelsman R, Arnold A, Carpenter TO. Surveillance for early detection of aggressive parathyroid disease: carcinoma and atypical adenoma in familial isolated hyperparathyroidism associated with a germline HRPT2 mutation. J Bone Miner Res. 2006; 21:1666–71.
25. Khadilkar KS, Budyal SR, Kasliwal R, Lila AR, Bandgar T, Shah NS. HRPT2- (CDC73) related hereditary hyperparathyroidism: a case series from Western India. Endocr Pract. 2015; 21:1010–6.
26. Kong J, Wang O, Nie M, Shi J, Hu Y, Jiang Y, Li M, Xia W, Meng X, Xing X. Familial isolated primary hyperparathyroidism/hyperparathyroidism-jaw tumour syndrome caused by germline gross deletion or point mutations of CDC73 gene in Chinese. Clin Endocrinol (Oxf). 2014; 81:222–30.
27. Korpi-Hyövälti E, Cranston T, Ryhänen E, Arola J, Aittomäki K, Sane T, Thakker RV, Schalin-Jäntti C. CDC73 intragenic deletion in familial primary hyperparathyroidism associated with parathyroid carcinoma. J Clin Endocrinol Metab. 2014; 99:3044–8.
28. Kutcher MR, Rigby MH, Bullock M, Trites J, Taylor SM, Hart RD. Hyperparathyroidism-jaw tumor syndrome. Head Neck. 2013; 35:E175–7.
29. Masi G, Barzon L, Iacobone M, Viel G, Porzionato A, Macchi V, De Caro R, Favia G, Palù G. Clinical, genetic, and histopathologic investigation of CDC73-related familial hyperparathyroidism. Endocr Relat Cancer. 2008; 15:1115–26.
30. Mizusawa N, Uchino S, Iwata T, Tsuyuguchi M, Suzuki Y, Mizukoshi T, Yamashita Y, Sakurai A, Suzuki S, Beniko M, Tahara H, Fujisawa M, Kamata N, et al. Genetic analyses in patients with familial isolated hyperparathyroidism and hyperparathyroidism-jaw tumour syndrome. Clin Endocrinol (Oxf). 2006; 65:9–16.
31. Moon SD, Park JH, Kim EM, Kim JH, Han JH, Yoo SJ, Yoon KH, Kang MI, Lee KW, Son HY, Kang SK, Oh SJ, Kim KM, et al. A Novel IVS2–1G>A mutation causes aberrant splicing of the HRPT2 gene in a family with hyperparathyroidism-jaw tumor syndrome. J Clin Endocrinol Metab. 2005; 90:878–83.
32. Panicker LM, Zhang JH, Dagur PK, Gastinger MJ, Simonds WF. Defective nucleolar localization and dominant interfering properties of a parafibromin L95P missense mutant causing the hyperparathyroidism-jaw tumor syndrome. Endocr Relat Cancer. 2010; 17:513–24.
33. Pazienza V, la Torre A, Baorda F, Alfarano M, Chetta M, Muscarella LA, Battista C, Copetti M, Kotzot D, Kapelari K, Al-Abdulrazzaq D, Perlman K, Sochett E, et al. Identification and functional characterization of three NoLS (nucleolar localisation signals) mutations of the CDC73 gene. PLoS One. 2013; 8:e82292.
34. Pimenta FJ, Gontijo Silveira LF, Tavares GC, Silva AC, Perdigão PF, Castro WH, Gomez MV, Teh BT, De Marco L, Gomez RS. HRPT2 gene alterations in ossifying fibroma of the jaws. Oral Oncol. 2006; 42:735–9.
35. Rekik N, Ben Naceur B, Mnif M, Mnif F, Mnif H, Boudawara T, Abid M. Hyperparathyroidism-jaw tumor syndrome: a case report. Ann Endocrinol (Paris). 2010; 71:121–6.
36. Sarquis MS, Silveira LG, Pimenta FJ, Dias EP, Teh BT, Friedman E, Gomez RS, Tavares GC, Eng C, De Marco L. Familial hyperparathyroidism: surgical outcome after 30 years of follow-up in three families with germline HRPT2 mutations. Surgery. 2008; 143:630–40.
37. Shibata Y, Yamazaki M, Takei M, Uchino S, Sakurai A, Komatsu M. Early-onset, severe, and recurrent primary hyperparathyroidism associated with a novel CDC73 mutation. Endocr J. 2015; 62:627–32.
38. Siu WK1, Law CY, Lam CW, Mak CM, Wong GW, Ho AY, Ho KY, Loo KT, Chiu SC, Chow LT, Tong SF, Chan AY. Novel nonsense CDC73 mutations in Chinese patients with parathyroid tumors. Fam Cancer. 2011; 10:695–9.
39. Starker LF, Akerström T, Long WD, Delgado-Verdugo A, Donovan P, Udelsman R, Lifton RP, Carling T. Frequent germ-line mutations of the MEN1, CASR, and HRPT2/CDC73 genes in young patients with clinically non-familial primary hyperparathyroidism. Horm Cancer. 2012; 3:44–51.
40. Sulaiman L, Haglund F, Hashemi J, Obara T, Nordenström J, Larsson C, Juhlin CC. Genome-wide and locus specific alterations in CDC73/HRPT2-mutated parathyroid tumors. PLoS One. 2012; 7:e46325.
41. Sulaiman L, Nilsson IL, Juhlin CC, Haglund F, Höög A, Larsson C, Hashemi J. Genetic characterization of large parathyroid adenomas. Endocr Relat Cancer. 2012; 19:389–407.
42. Takeuchi T, Yoto Y, Tsugawa T, Kamasaki H, Kondo A, Ogino J, Hasegawa T, Yama N, Anan S, Uchino S, Ishikawa A, Sakurai A, Tsutsumi H. An adolescent case of familial hyperparathyroidism with a germline frameshift mutation of the CDC73 gene. Clin Pediatr Endocrinol. 2015; 24:185–9.
43. Veiguela B, Isidro ML, Jorge S, Ruano B. An uncommon cause of hypercalcemia: synchronous carcinoma of two parathyroids in the context of hyperparathyroidism-jaw tumor syndrome. Endocrinol Nutr. 2010; 57:391–3.
44. Villablanca A, Calender A, Forsberg L, Höög A, Cheng JD, Petillo D, Bauters C, Kahnoski K, Ebeling T, Salmela P,

Richardson AL, Delbridge L, Meyrier A, et al. Germline and *de novo* mutations in the HRPT2 tumour suppressor gene in familial isolated hyperparathyroidism (FIHP). J Med Genet. 2004; 41: e32.

45. Wang O, Wang C, Nie M, Cui Q, Guan H, Jiang Y, Li M, Xia W, Meng X, Xing X. Novel HRPT2/CDC73 gene mutations and loss of expression of parafibromin in Chinese patients with clinically sporadic parathyroid carcinomas. PLoS One. 2012; 7:e45567.

46. Yamashita Y, Akiyama T, Mizusawa N, Yoshimoto K, Goto M. A case of hyperparathyroidism-jaw tumour syndrome found in the treatment of an ossifying fibroma in the maxillary bone. Int J Oral Maxillofac Surg. 2007; 36:365–9.
